# The risk for post-thrombotic syndrome of subjects with deep vein thrombosis in an Indonesian referral hospital: a retrospective cohort study

**DOI:** 10.1186/s12959-023-00482-7

**Published:** 2023-04-05

**Authors:** Farieda Ariyanti, Lugyanti Sukrisman, Dono Antono, Kuntjoro Harimurti

**Affiliations:** 1 Department of Internal Medicine, Faculty of Medicine, Universitas Indonesia, Cipto Mangunkusumo General Hospital, Jakarta, Indonesia; 2 Division of Hematology - Medical Oncology, Department of Internal Medicine, Faculty of Medicine, Universitas Indonesia, Cipto Mangunkusumo General Hospital, Jakarta, Indonesia; 3 Cardiovascular Division- Medical Oncology, Department of Internal Medicine, Faculty of Medicine, Universitas Indonesia, Cipto Mangunkusumo General Hospital, Jakarta, Indonesia; 4 Clinical Epidemiology Unit, Department of Internal Medicine, Faculty of Medicine, Universitas Indonesia, Cipto Mangunkusumo General Hospital, Jakarta, Indonesia

**Keywords:** Deep vein thrombosis, Post-thrombotic syndrome, Villalta score

## Abstract

**Background:**

Post-thrombotic syndrome (PTS) is a complication of deep vein thrombosis (DVT) and affects 20–40% of DVT subjects. The risk factor of PTS after DVT is difficult to determine. We aimed to evaluate the incidence of PTS after 3 months of DVT diagnosis and to determine the risk of PTS.

**Methods:**

A retrospective cohort study of subjects who developed DVT confirmed by Doppler ultrasound in Cipto Mangunkusumo Hospital from April 2014 until June 2015. The presence of PTS was assessed after 3 months of completed DVT treatment using the Villalta score. Risk factors for PTS were evaluated from medical records.

**Results:**

There were 91 subjects with DVT with mean age of 58 years. 56% were female. It was dominated by subjects aged ≥ 60 years (45.1%). Hypertension (30.8%) and diabetes mellitus (26.4%) were the major comorbidities in this study. Deep vein thrombosis occurred commonly in unilateral side (79.1%), proximal localization (87.9%), and unprovoked DVT (47.3%). The cumulative incidence of PTS after DVT was 53.8%, 69% of subjects had mild PTS. Heaviness of the leg (63.2%) and edema (77.5%) were the most common symptoms.

**Results:**

There were 91 subjects with DVT with mean age of 58 years. 56% were female. It was dominated by subjects aged ≥ 60 years (45.1%). Hypertension (30.8%) and diabetes mellitus (26.4%) were the major comorbidities in this study. Deep vein thrombosis occurred commonly in unilateral side (79.1%), proximal localization (87.9%), and unprovoked DVT (47.3%). The cumulative incidence of PTS after DVT was 53.8%, 69% of subjects had mild PTS. Heaviness of the leg (63.2%) and edema (77.5%) were the most common symptoms. Significant risk factors for PTS were unprovoked DVT (adjusted RR 1.67; 95%CI: 1.17–2.04; p = 0.01) and female gender (adjusted RR 1.55; 95%CI: 1.03–1.94; p = 0.04). Age, body mass index, thrombus location, immobilization, malignancy and surgery was not associated with PTS.

**Conclusion:**

We conclude that 53.8% of subjects suffered PTS after 3 months of DVT. Unprovoked DVT and female gender were significant risk factors for PTS.

## Introduction

Post-thrombotic syndrome (PTS) is the most common chronic complication of deep vein thrombosis (DVT). PTS affected 20–40% of patient with DVT, and 5–10% of subjects develop severe symptoms of PTS, which could decrease the quality of life of the subjects [1, 2].

Clinical manifestations of PTS are edema, heaviness in lower extremities, extremity pain, and skin lesions such as eczema, hyperpigmentation, lipodermatosclerosis, and skin ulcer [3]. One of two to three subjects diagnosed with DVT will develop PTS within two years with clinical variation, from mild symptoms (skin pigmentation, telangiectasia, mild pain, and edema) to severe symptoms (chronic pain, severe edema, and leg ulcer) [4].

Based on recent studies, risk factors for the patient with DVT to develop PTS are patient’s characteristics on the first onset of DVT (old age, female gender, high body mass index or obesity, thrombophilia, and thrombus location in iliac and femoral vein), recurrent DVT in the ipsilateral site, duration and the intensity of oral anticoagulant use, vein abnormalities in Doppler ultrasound (residual thrombosis in a patient with recurrent DVT, or vein reflux), and increase of D-dimer (> 500 ng/mL) after three weeks withdrawal of anticoagulant therapy [3, 5, 6].

Given that insufficient data on PTS are available in Indonesia, we aimed to evaluate the PTS incidence after 3 months of completed treatment of DVT and to determine the relative risk of PTS of each predictor in DVT subjects in Cipto Mangunkusumo General Hospital, Jakarta.

## Methods

### Subjects and study design

This was a retrospective cohort study. Subjects were patients aged 18 years and older with confirmed lower extremity DVT by Doppler ultrasound in the Integrated Heart Center Cipto Mangunkusumo General Hospital Jakarta from January 2010 – June 2015. Diagnosis was made based on duplex ultrasound performed by experienced cardiology and vascular medicine specialists. Diagnosis of DVT was confirmed if the ultrasound showed non-compressible vein on 2 dimensional B-mode and no flow on color or spectral Doppler imaging. Determination of PTS was made after 3 months of treatment cessation. The exclusion criteria were recurrent DVT at subject recruitment, confirmed by Doppler ultrasonography; fluid overload caused by congestive heart failure or chronic kidney disease. Initial anticoagulant therapy during admission for episode of DVT was recorded, which consist of parenteral (unfractionated heparin, low molecular weight heparin, or fondaparinux) during acute phase, followed by warfarin, rivaroxaban, or others starting at DVT diagnosis until discharge and outpatient period, or without therapy due to contraindication to anticoagulant therapy.

The subjects were contacted by phone and asked to provide written informed consent. Those who were willing to join this study were asked to do in-person interview and a series of physical and ultrasonography examination during April 2014 until June 2015. The Villalta score was obtained during this encounter. Risk factors for PTS were obtained from medical records and confirmed by the subjects during the evaluation. All subjects during the study period were included in the study (total sampling).

The subjects are asked to discontinue any painkillers or the use of elastic compression stockings a day prior to in-person interviews and physical examinations. The subjects filled out the questionnaire about acquired risk factors of DVT. To avoid bias, the assessor of symptoms and signs of PTS was a resident who was not involved in patient care and unaware of previous conditions the subjects had.

The subjects were divided into groups of unprovoked (idiopathic) DVT and provoked DVT as the subjects had one or more of the following risk factors: trauma, surgery, central catheterization in femoral vein, malignancy, immobilization.

PTS was assessed using the Villalta PTS scale by asking 5 symptoms and 6 signs. Each symptom and sign are rated as 0 (absent), 1 (mild), 2 (moderate), 3 (severe), except for ulcer which is noted as present or absent. The points are summed into a total score. PTS presents if the total score is ≥ 5 or the presence of ulcer in the same leg of the previous DVT, regardless of the total score. PTS is categorized as mild (score 5–9), moderate (score 10–14), and severe (score ≥ 15 or the presence of ulcer).

Proximal DVT was defined as thrombus located in popliteal or more proximal veins (either popliteal, femoral, or iliac veins), whereas distal DVT was defined as thrombus located in tibial veins (either peroneal, tibial, gastrocnemius, or soleal veins).

The body mass index (BMI) was categorized based on the World Health Organization 2000 criteria for adults as underweight (BMI < 18.5 kg/m^2^), normal (BMI 18.5–24.9 kg/m^2^), overweight (BMI 25-29.9 kg/m^2^), and obese (BMI ≥ 30 kg/m^2^).

### Statistical analyses

The data were presented as frequency and mean ± standard deviation (SD). The cumulative incidence of PTS was calculated as the number of subjects with PTS divided by the number of participating subjects. The differences between the two groups were determined using the chi-square test, with p-value < 0.05 indicate a statistical significance difference. The relative risk was measured by risk ratios (RRs) and 95% confidence intervals (CIs) which was calculated with the risk calculation menu from the software along with the Chi square test. The RRs predict the risk of developing PTS in the presence of the predictor compared to the absence of the predictor. Multivariate analysis was established using Enter method of binomial logistic regression. Variables with p value < 0.20 in bivariate analysis were included. The obtained risk was generated by the software in the form of adjusted Odds Ratio, which was calculated to adjusted RR using statistical formula for corrected RR: [7]

$$RR=\frac{OR}{\left(1-{P}_{o}\right)+({P}_{o}\times OR)}$$

All statistical analyses were performed with SPSS version 22.

## Results

From 846 subjects with clinically suspected DVT during January 2010 – June 2015, 236 had incomplete data, 89 subjects had no thrombus found on Doppler ultrasonography, and 32 had thrombosis of the upper extremity. Of the 489 subjects with confirmed DVT of the leg, 234 deceased before they could participate, 82 could not be contacted, 71 lived outside Jakarta, 6 refused to participate in this study, and 5 met the exclusion criteria, leaving only 91 participating subjects in this study. Most of the deceased subjects had DVT and cancer.

### Clinical characteristics of the subjects

The characteristics of the 91 subjects are summarized in Table 1. There were 40 males (44%) and 51 females (56%), age ranged from 24 to 85 years, and 45.1% were subjects aged 60 years and older. The majority of the subjects had normal and overweight BMI (41.8% and 34.1% respectively). The comorbidities in the subjects were hypertension (30.8%), diabetes mellitus (26.4%), coronary heart disease (18.7%), chronic kidney disease (18.7%), knee osteoarthritis (14.3%), stroke (12.1%), thrombophilia (9.9%), chronic heart failure (8.8%), autoimmune disease (6.6%), spine disease (5.5%) and major thalassemia (2.1%). Only 27 of 91 subjects (29.7%) wore elastic compression stockings after the DVT.

**Table 1:** Clinical characteristics of subjects with DVT (n = 91)

Subject’s Characteristics	n (%)
Gender, n(%)	
Male Female	40 (44) 51 (56)
**Age (years), mean (SD)**	58 (13.8)
**Age (years), n(%)**	
18–29 30–44 45–59 ≥ 60	4 (4.4) 16 (17.6) 30 (33) 41 (45.1)
**BMI (kg/m** ^**2**^ **), mean (SD)**	25.26 (4.8)
**BMI (kg/m** ^**2**^ **), n(%)**	
< 18,5 18,5–24,9 25–29,9 ≥ 30	9 (9.9) 38 (41.8) 31 (34.1) 13 (14.3)
**Morbidity, n(%)**	
Hypertension Diabetes mellitus Coronary heart disease Chronic kidney disease Knee osteoarthritis Stroke Thrombophilia Chronic heart failure Autoimmune disease Spine disease Major thalassemia	28 (30.8) 24 (26.4) 17 (18.7) 17 (18.7) 13 (14.3) 11 (12.1) 9 (9.9) 8 (8.8) 6 (6.6) 5 (5.5) 2 (2.1)

SD, standard deviation; BMI, body mass index

### DVT characteristics and risk factors of the subjects

The DVT characteristics from 91 subjects in this study were categorized as the amount of extremity affected by DVT, the number of veins affected by DVT, and thrombus location (Table 2). Unilateral thrombosis was found in 20.9% of subjects. There were 87.9% subjects with proximal thrombus, 1.1% had distal thrombus, and 11% have proximal and distal thrombus.

Forty-three of 91 subjects (47.3%) had unprovoked (idiopathic) DVT. The risk factors of provoked DVT were immobilization (18.7%), malignancy (17.6%), central vein catheterization in femoral vein (7.7%), surgery (4.4%), and trauma (4.4%). Most of the subjects (89%) had only one risk factor of DVT. The majority of the patients received unfractionated heparin (47.3%) and warfarin (25.3%). Most of them had more than 3 months of anticoagulation treatment.

**Table 2:** Clinical characteristic of DVT in 91 subjects

Characteristics of DVT	n (%)
**Extremity affected by DVT**	
Unilateral	72 (79.1)
Bilateral	19 (20.9)
**Number of veins affected by DVT**	
Single	45 (49.5)
Multiple	46 (50.5)
**Thrombus location**	
Proximal only	80 (87.9)
Distal only	1 (1.1)
Proximal-distal	10 (11)
**Risk factor of DVT**	
Unprovoked DVT (idiopathic)*	43 (47.3)
Immobilization	17 (18.7)
Malignancy	16 (17.6)
Central vein catheterization in femoral vein	7 (7.7)
Surgery	4 (4.4)
Trauma	4 (4.4)
**Number of DVT risk factor**	
One risk factor	81 (89)
More than one risk factor	10 (11)
**Anticoagulant Therapy**	
Unfractionated heparin/warfarin	43 (47.3)
Low molecular weight heparin/warfarin	5 (5.5)
Fondaparinux/warfarin	6 (6.6)
Warfarin only	23 (25.3)
Rivaroxaban	8 (8.8)
Others (acetylsalicylate, herbal medicine)	2 (2.2)
No therapy	4 (4.4)
**Anticoagulant Duration**	
< 3 months	31 (36.5)
3–6 months	20 (23.5)
> 6 months	35 (40)

DVT, deep vein thrombosis; * Subjects without risk factors for provoked DVT: immobilization, malignancy, central vein catheterization in femoral vein, surgery, trauma

### The cumulative incidence and severity of PTS

The cumulative incidence of PTS was 53.8% (95% CI 35.1–62.9). Based on its severity, it was mild in 69% of subjects, moderate in 29% of subjects, and severe in 2% of subjects. Symptoms and signs of PTS reported from the subjects are shown in Table 3. Each subject could report more than one symptom of PTS. The most common PTS symptom reported by 34 subjects (63.2%) was the heaviness of the leg, whereas the most common sign reported by 38 subjects (77.5%) was leg swelling. None of our subjects reported having active leg ulcers.

**Table 3:** Post thrombotic symptoms and signs in 91 subjects

Symptoms and signs	n (%)
**Symptoms**	
Heaviness	34 (63.2)
Pain	28 (57.1)
Cramp	28 (57.1)
Paresthesia	24 (48.9)
Pruritus	18 (36.7)
**Signs**	
Leg swelling	38 (77.5)
Hyperpigmentation	34 (69.4)
Telangiectasia	28 (57.1)
Pain on calf compression	12 (21.1)
Skin induration	9 (18.4)
Redness	7 (14.9)

### Risk factors of PTS

The characteristics and risk factors of DVT were analyzed to find the relative risk (RR) of PTS (Table 4**)**. From the bivariate analysis, unprovoked DVT was a significant risk factor for PTS (RR 1.62, 95% CI: 1.01–2.40, p = 0.01). PTS developed in 24 of 41 (58.5%) subjects aged 60 years and older, as compared to 25 of 50 (50%) subjects aged 18–59 years, leading to a 1.17-fold increased risk of developing PTS, despite not statistically significant (RR 1.17, 95% CI: 0.80–1.71, p = 0.527). The risk of PTS was 1.48-fold higher in females than in males (RR 1.48, 95% CI: 0.97–2.42, p = 0.06). Proximal and distal DVT was associated with a 1.35-fold increased risk of development of PTS compared to either proximal or distal DVT alone (RR 1.35, 95% CI: 0.85–2.13, p = 0.331). Body mass index was not associated with the incidence of PTS (RR 1.02, 95% CI: 0.58–1.72, p = 1.00). Moreover, immobilization, malignancy and surgery were not associated with PTS. Multivariate analysis showed that unprovoked DVT and gender were significant factors for PTS (adjusted RR [aRR] 1.67, 95% CI: 1.17–2.04; p = 0.01 and aRR 1.55; 95% CI: 1.03–1.94; p = 0.04, respectively)

**Table 4:** Risk factor of PTS after 3 months of DVT

Variable	With PTS (n = 49)	Without PTS (n = 42)	p-value (chi-square)	RR (95% CI)	p value	Adjusted RR (95% CI)
**Age (years), n (%)**						
≥ 60	24 (58.5)	17 (41.5)	0.53	1.17 (0.80–1.71)		
18–59	25 (50)	25 (50)				
**Gender, n (%)**						
Female	32 (62.7)	19 (37.3)	0.06	1.48 (0.97–2.42)	0.04	1.55 (1.03–1.94)
Male	17 (42.5)	23 (57.5)				
**BMI (kg/m** ^**2**^ **), n (%)**						
Obesity	7 (53.8)	6 (46.2)	1.00	1.02 (0.58–1.72)		
Not obesity	42 (53.8)	36 (46.2)				
**Thrombus Location, n(%)**						
Proximal-distal	7 (70)	3 (30)	0.33	1.35 (0.85–2.13)		
Proximal only or distal only	42 (51.9)	39 (48.1)				
**Unprovoked DVT**						
Yes	29 (67.4)	14 (32.6)	0.01	1.62 (1.01–2.40)	0.01	1.67 (1.17–2.04)
No	20 (41.7)	28 (58.3)				
**Immobilization, n(%)**						
Yes	9 (52.9)	8 (47.1)	0.93	0.98 (0.60–1.61)		
No	40 (54.1)	34 (45.9)				
**Malignancy, n(%)**						
Yes	7 (43.8)	9 (56.2)	0.37	0.78 (0.243–1.41)		
No	42 (56)	33 (44)				
**Surgery, n(%)**						
Yes	2 (50)	2 (50)	0.88	0.93 (0.34–2.51)		
No	47 (54)	40 (46)				
**Trauma, n(%)**						
Yes	1 (25)	3 (75)	0.24	0.45 (0.08–2.5)		
No	48 (55.2)	39 (44.8)				

CI, confidence interval; DVT, deep vein thrombosis; PTS, post-thrombotic syndrome; RR, relative risk

## Discussion

We found that the incidence of PTS was 53.8% of subjects with DVT; the majority of our subjects presented the mild form of PTS (Villalta score 5–9). Tick et al. found the cumulative incidence of PTS after 3 months was 46% and Schulman et al. showed that PTS was seen in 56% of subjects even after 10 years [8, 9].

Most of our subjects (63.2%) complained about heaviness in the leg as a sign of PTS. This is relevant to a study by Tick et al. [10]. Moreover, leg swelling (edema) was the most prominent sign (77.5%) in our study. Patients who have early leg swelling are more likely to have residual thrombosis resulting in a higher risk of recurrent venous thromboembolism (VTE) [11, 12].

A small group of our subjects (9.9%) had thrombophilia, including the presence of anticardiolipin antibody (ACA) IgG; confirmed antiphospholipid syndrome (APS); sticky platelet syndrome (SPS); deficiencies of protein C/protein S or antithrombin III. Due to the small numbers of subjects with thrombophilia, we could not analyze the relative risk of this group. In a cohort study of 310 subjects with thrombophilia by Zutt et al., protein S deficiency and Factor V Leiden were more prevalent significantly in the PTS group (p = 0.035 and p = 0.003 respectively) [13]. Results of the study are still conflicting regarding the association of thrombophilia and PTS [14].

Unprovoked DVT was a significant risk factor for PTS. Those who experienced provoked VTE had transient risk factor of VTE. In contrast to that, those who developed unprovoked VTE had no identifiable risk factor or provoking event which could not be controlled. Therefore, the risk of VTE recurrence in patients with unprovoked VTE was higher, approximately twice that of provoked VTE. Recurrent VTE might lead to thrombus injuring venous valves in deep veins of the leg, resulting in leg edema, leg pain, skin ulceration, and skin hyperpigmentation. These are the signs and symptoms of PTS [15, 16]. However, it is often challenging to differentiate the clinical presentation of recurrent VTE and PTS when the symptoms is subacute [17]. Doppler ultrasonography was evaluated in every patients in this study to ensure those who included in this study had no recurrent DVT.

In this study, we found that women are at a higher risk of developing PTS than men, with a cumulative incidence after 3 months of 62.7% vs. 42.5% (RR 1.48, 95% CI 0.97–2.42, p = 0.06; aRR 1.55; 95% CI 1.03–1.94; p = 0.04). The increased incidence of PTS has been described in both men and women [10, 18]. However, the underlying mechanisms are still poorly understood; they might include anatomical, mechanical, and hormonal aspects [19].

We found that older age (≥ 60 years old) and proximal-distal localization of the thrombus appear to have an increased risk, yet not significant, of developing PTS. Some studies also reported the increased risk of PTS in the elderly [1, 20]. Stain et al. described that in advancing age, the odd ratio for PTS development was 1.2 for every ten-year increase [18]. The increased incidence of thrombotic in the elderly in this study might be explained by the hypercoagulability state and advanced sclerotic changes in the vascular wall [21]. Moreover, hormonal factors, inflammatory response to thrombus, and calf pump function may differ by age [19]. Immobilization, malignancy and surgery were not associated with PTS. These are known risk factors for recurrence of DVT, but they do not contribute to increased risk of PTS.

Our data showed that, although non statistically significant, PTS occurred more frequently in subjects who had proximal and distal DVT rather than in subjects with proximal or distal DVT alone. Gabriel et al. highlighted the highest incidence of PTS in subjects who had proximal and distal DVT, followed by proximal DVT, and distal DVT at the last [22]. Stain et al. revealed that proximal DVT, compared to distal DVT, conferred up to twofold increased risk of PTS (OR 2.0, 95% CI 1.3–3.1) [18]. Resolution of the proximal thrombus occurs in a slower rate compared to the distal thrombus [23]. It might explain why the proximal localization of DVT was significantly related to a more severe PTS [24]. Nevertheless, Ziegler et al. found no significant association between the site of the DVT and the occurrence of the PTS [25]. In addition, despite 48.4% of the subjects being overweight/obese, we found no association between increased body weight and PTS development.

In this study, most of the patients received unfractionated heparin, followed with warfarin. In the past, the only option for VTE treatment was vitamin K antagonist (warfarin) with initial heparin therapy. Since 2009, direct oral anticoagulant (DOAC), such as rivaroxaban, has gained popularity for the treatment of VTE because it is effective, has few potential drug interactions, and does not require routine laboratory monitoring. According to CHEST guideline, it is recommended to complete a 3-month phase of anticoagulation. Furthermore, patients with provoked VTE or transient risk factor necessiting 3–6 months of anticoagulation for secondary prophylaxis. Meanwhile, patients with unprovoked VTE are recommended to receive prolonged anticoagulation because of higher VTE recurrence risk [26]. Most of our patients received more than 3 months of anticoagulation treatment. This might be related to higher proportion of unprovoked VTE in our patients.

Most of our subjects (70.3%) did not use stockings as it is not covered by Government insurance. Wearing elastic compression stockings in daily life can be burdensome for the subjects. Indeed, it is uncomfortable, difficult to put on or take off, unappealing, and expensive [1]. In 2014, a guideline published by American Heart Association (AHA) recommended the use of elastic stockings to reduce symptomatic leg swelling [27]. It is hypothesized that elastic stockings may assist the muscle pump and counterbalance venous hypertension as a result of valve damage and/or venous obstruction that occurs after a thrombotic episode; thus it could play a role in preventing PTS. Yet, the benefits of the long-term use of elastic stockings are still unclear [1, 28]. For this reason, in 2016, the American College of Chest Physicians (ACCP) guideline does not recommend routine use of elastic stockings after DVT for PTS prevention [29]. Instead, elastic compression stocking may be helpful to counteract the edema formation.

We used a validated scale (Villalta score), which has high reproducibility, to diagnose PTS. Bias was avoided by using an assessor of PTS who was not involved in patient care and unaware of previous conditions of the subjects.

There are some limitations in this study. First, our sample size was quite small due to limited number of accessible patients. Second, we retrieved the data retrospectively hence numerous of the subjects had to be excluded – the majority of the subjects deceased from cancer or other diseases, could not be contacted, or lived outside Jakarta. Unfortunately, the data from the deceased patients was not sufficient to include them in this study. Since this study requires active monitoring of the participants at the beginning of the cohort by Doppler ultrasound and Villalta score, data extraction solely by medical records was not possible. Although this method reduced the sample size, the possibility of bias due to recurrent DVT diagnosed as PTS or recall bias in the Villalta score was minimized. Third, although all patients were given anticoagulants, we could not analyze the intensity of the treatment to the risk of PTS in the subjects. Current study might serve as pilot study for further studies in the future. Further study with prospective cohort is needed to give more information about the risk of PTS.

## Conclusion

From the results of this study, we conclude that PTS occurred in 53.8% of subjects after 3 months of treatment cessation for DVT. Unprovoked DVT and female gender was a significant risk factor for PTS. Age, body mass index, thrombus location, immobilization, malignancy and surgery was not associated with PTS.

## Data Availability

The datasets used in the current study are available from the corresponding author on reasonable request.

## Abbreviations

ACA: Anticardiolipin antibody
ACCP: American College of Chest Physicians
AHA: American Heart Association
APS: Antiphospholipid syndrome
BMI: Body mass index
CI: Confidence interval
DVT: Deep vein thrombosis
PTS: Post-thrombotic syndrome
RR: Risk ratio
SD: Standard deviation
SPS: Sticky platelet syndrome
VTE: Venous thromboembolism
